# Supplementary material for: A Real-World Comparative Analysis of Atezolizumab Plus Bevacizumab and Transarterial Chemoembolization Plus Radiotherapy in Hepatocellular Carcinoma Patients with Portal Vein Tumor Thrombosis
Source: Cancers (Basel). 2023 Sep 4;15(17):4423. doi: 10.3390/cancers15174423 (PMC10486735; doi:10.3390/cancers15174423)
Supplement: Supplementary file 1 [file cancers-15-04423-s001.zip › cancers-2538515-supplementary.pdf]

cancers

MDPI

Supplementary Materials

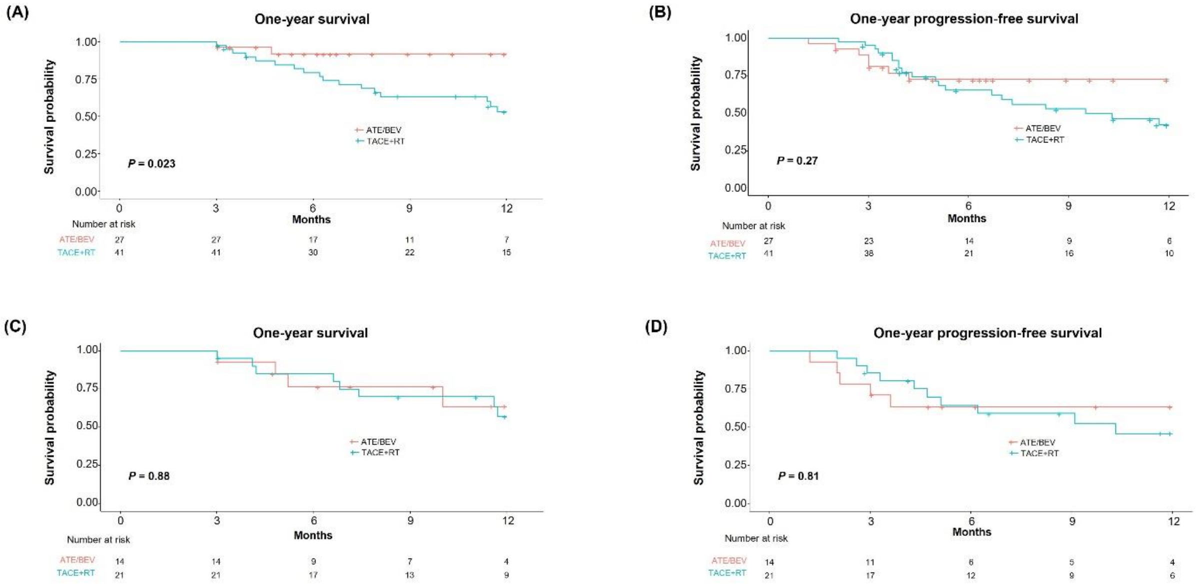

Figure S1. One-year survival and progression-free survival in patients with (A,B) multiple intrahepatic HCC, and (C,D) unilobar intrahepatic HCC without VP4 PVTT. HCC, hepatocellular carcinoma; VP4 PVTT, major portal vein tumor thrombus.
